# Activation of TLR4-Mediated NF*κ*B Signaling in Hemorrhagic Brain in Rats

**DOI:** 10.1155/2009/473276

**Published:** 2010-01-31

**Authors:** Weiyu Teng, Lishu Wang, Weishuang Xue, Chao Guan

**Affiliations:** ^1^Department of Neurology, The First Affiliated Hospital, China Medical University, Shenyang 110001, China; ^2^Department of Otorhinolaryngology, The First Affiliated Hospital, China Medical University, Shenyang 110001, China

## Abstract

Inflammation and immunity play a crucial role in the pathogenesis of Intracerebral hemorrhage (ICH). Toll-like receptor 4- (TLR4-) mediated nuclear factor kappa-B (NF*κ*B) signaling plays critical roles in the activation and regulation of inflammatory responses in injured brain. However, the involvement of TLR4-mediated NF*κ*B signaling in the pathogenesis of ICH remains unknown. The present study was to evaluate the temporal profile of the expression of TLR4 and the activation of TLR4-mediated NF*κ*B signaling in brain tissues of Wistar rats after ICH. TLR4 mRNA and protein, the phosphorylation of inhibitors of kappa B (p-I*κ*B*α*), and the activity of NF*κ*B were examined in hemorrhagic cerebral tissue by Rt-PCR, Western blots, immunohistochemistry staining, and EMSA. Compared with saline control, the TLR4 mRNA and protein significantly increased starting at 6 hours after ICH, peaked on the 3rd day after ICH, and then decreased but still maintained at a higher level on the 7th day after ICH (*P* < .05). The level of p-I*κ*B*α* and the activity of NF*κ*B also increased in the brain after ICH compared with saline control. The present study firstly suggests that TLR4-mediated NF*κ*B signaling participates in the pathogenesis of ICH, which may become a therapeutic target for ICH-induced brain damage.

## 1. Introduction

 Intracerebral hemorrhage (ICH) represents 10%–15% of all strokes and occurs with increasing frequency as a complication of the thrombolytic treatment of ischemic stroke, which is associated with high mortality and severe disability in survivors [1, 2]. Local brain tissue deformation induces primary brain damage after ICH; more importantly, the excitotoxicity and inflammatory responses in the brain tissue induced by ICH result in further brain damage following ICH [3]. Both innate and adaptive immunological/inflammatory mechanisms have been clearly shown to participate in the progression of brain injury after ischemic stroke. Nevertheless, how the inflammatory responses in ICH are triggered and regulated remains to be elucidated. 

 TLRs are a family of transmembrane proteins and act as signal transduction molecules [4]. Activation of TLRs recruits downstream signaling proteins and results in transcription of genes encoding inflammation-associated molecules and cytokines [5, 6]. For example, combined with specific ligand, the activation of TLR4 induces the phosphorylation of inhibitor of kappa-B (I*κ*B) and dissociates I*κ*B from NF-kappa-B (NF*κ*B). Then, NF*κ*B trans-locates into the nucleus and activates and regulates the transcription of genes related to inflammatory responses [5, 6]. TLRs play a critical role in the induction of immunological/inflammatory responses and are implicated in several CNS diseases, such as infectious disease, autoimmune disease, and ischemic brain injury [7, 8]. Accumulating evidences demonstrate that TLR4, the first mammalian TLR recognized, contributes to the neuronal death, blood brain barrier damage, brain edema, and inflammatory responses in the brain injury induced by ischemia [8, 9]. However, little is known about TLR4 in the brain after ICH. In the present study, we detected the transcription of TLR4 gene and the activation of TLR4-mediated signaling in the rat brains subjected to experimental ICH. Our data firstly provided the knowledge about the distribution of TLR4 and the temporal profile of the activation of TLR4-mediated signaling in rat brain tissue after ICH.

## 2. Methods

### 2.1. Animals

Ninety Wister rats (male, body weight: 250 g to 300 g) were provided by Experimental Animal Center, China Medical University. The rats were assigned randomly to three groups: normal group (N, *n* = 10, without any treatment), normal saline control (S, *n* = 40, intra-cerebral injection of normal saline as control) and intra-cerebral hemorrhage group (ICH, *n* = 40). The experimental protocol used for animals was approved by the Animal Care and Use Committee, China Medical University. The rats were subjected to intra-cerebral injection of normal saline or autologous whole blood and were sacrificed on 6 hours, 1 day, 3 days and 7 days after the injection, respectively. Five rats in each group at each time point were sacrificed by transcardially perfusion of normal saline followed by 4% buffered Para formaldehyde (pH = 7.4). The brains were removed, postfixed, embedded in paraffin, and cut into sections (7 *μ*m) as described previously [8]. Five rats in each group at each time point were sacrificed by over dose injection of Ketamine. The brains were removed and stored at minus 80°C for RT-PCR and Western Blots.

### 2.2. Induction of Intracerebral Hemorrhage

Since most intracerebral hemorrhages occur in the basal ganglia clinically, in the present study, rat model of ICH was induced in left basal ganglia. Rats were anesthetized with intra-peritoneal sodium pentobarbital (60 mg/kg). The body (rectal) temperature was maintained at 37°C during the surgery using a feedback-controlled heating system. The rats were positioned in a stereotactic frame, and the scalp was incised along the midline. Using a sterile technique, a 1 mm burr hole was opened in the skull on the left coronal suture 3 mm lateral to the midline. A blunt 26-gauge needle was inserted into the left basal ganglia under stereotactic guidance (coordinates: 0.2 mm anterior, 6.0 mm ventral, and 3.0 mm lateral to the midline). Then, a 75 *μ*L of autologous whole blood was infused at a rate of 20 *μ*L/min with the use of a microinfusion pump. After the completion of the infusion, the needle was withdrawn quickly, cyanoacrylate glue was placed around the burr hole, and the skin incision was closed with sutures. For the normal saline control rats, only 75 *μ*L normal saline was injected.

### 2.3. Detection of TLR4 mRNA by the Method of RT-PCR

Briefly, total RNA samples were isolated from peri-hemorrhage regions in left basal ganglia using Simply *P* Total RNA isolation Kit (BioFlux, HZ, China) according to the manufacturer's instructions. The quantity of total RNA was determined on a Nanodrop (ND-1000 Spectrophotometer) by measuring optical density at A_260_ and A_280_ nm wavelength. Then, the concentrations of mRNA were adjusted to 1.0 g/mL. RNA was reverse-transcribed into cDNA in 20 *μ*L reaction system using Superscript First-Strand Synthesis Kit for RT-PCR (promega Inc) under conditions described by the supplier. One microliter of cDNA was used to amplify the TLR4 gene fragment with 1× PCR buffer, 1.5 mM MgCl_2_, 200 *μ*M of each dNTP, 200 *μ*M of primers, and 2u Taq DNA polymerase. The house keep gene (*β*-actin) was used as an internal control. The PCR primer sequences were designed according to the TLR4 and *β*-actin gene sequences reported in GenBank. TLR4 (145 bp): forward: 5′-AGCTTTGGTCAGTTGGCTCT-3′; reverse: 5′-CAGGATGACACCATTGAAGC-3′; *β*-actin (701 bp): forward: 5′-GCCAACCGTGAAAAAGATG-3′; reverse: 5′-CCAGGATAGAGCCACCAAT-3′. Reaction cycles were performed at the following condition: 94°C 2 min, 94°C 40 s, 55°C 40 s, and 72°C 60 s. The reaction was stopped after 31 cycles. PCR products were electrophoresed through agarose gel. Image Acquisition and Analysis software were used to determine band densities. The abundance of TLR4 mRNA was expressed as the ratio of TLR4 mRNA to *β*-actin mRNA.

### 2.4. Immunohistochemistry (IHC)

The IHC staining for TLR4 was performed as described previously [8]. The primary antibody employed was rabbit anti-TLR4 (WuHan, BA1717). The biotinylated secondary antibody and antibody-biotin-avidin-peroxidase complexes (ABC reagent, SC-2018) were obtained from Santa Cruz Biotechnology Inc. (Santa Cruz, CA). Slides processed without primary antibodies served as negative controls.

### 2.5. Western Blots

Cellular proteins were prepared from ICH cerebral hemispheres, electrophoresed with SDS-polyacrylamide gel and transferred onto Hybond ECL membranes (Amersham Pharmacia, Piscataway, NJ). The ECL membranes were incubated with the appropriate primary antibody (WuHan, BA1717) followed by incubation with peroxidase-conjugated secondary antibodies (Golden Bridge, PV-6001). The signals were detected with the ECL system (Amersham Pharmacia). The same membranes were probed with anti-GAPDH (glyceraldehyde-3-phosphate dehydrogenase, Biodesign, Saco, Maine) after being washed with stripping buffer. The signals were quantified by scanning densitometry and computer-assisted image analysis.

### 2.6. Electrophoretic Mobility Shift Assay (EMSA)

Nuclear proteins were isolated from brain tissue. Briefly, after removal of supernatant (cytoplasmic extract), the pellets were resuspended in nuclear extraction reagent. The tubes containing pellets were put on vortex for 15 seconds every 10 minutes, for a total of 40 minutes. The tubes were centrifuged at maximum speed (~16,000 × g) in a micro centrifuge for 10 minutes. The supernatants (nuclear extract) were transferred to a clean prechilled tube and stored at −80°C until use. EMSA was performed as described previously [8]. NF*κ*B binding activity was examined in a 15 *μ*L binding reaction mixture containing 15 *μ*g of nuclear proteins and 35 fmol [*γ*-^32^P] labeled double-stranded NF*κ*B consensus oligonucleotide. A super shift assay using antibodies to P65 and P50 was performed to confirm NF*κ*B binding specificity as previously described.

### 2.7. Statistical Analysis

All data were presented as mean ± SEM. The measurements were analyzed by one-way analysis of variance (ANOVA) or Student's *t*-test. The *P* < .05 level of probability was used as the criteria of significance.

## 3. Results

### 3.1. TLR4 mRNA Increased in Brain Tissue after ICH

**Figure 1:**
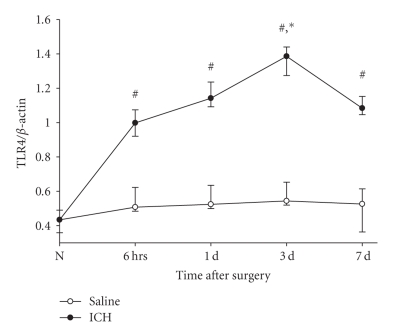
*The temporal changes of TLR4 mRNA in brain tissue after ICH*. TLR4 mRNA levels were detected by Rt-PCR. The abundance of TLR4 mRNA was expressed as the ratio of TLR4 mRNA to *β*-actin mRNA. As shown in Figure 1, the low level of TLR4 mRNA was observed in normal rat brain tissue. In the saline control group, the TLR4 mRNA slightly increased but was no significantly different from normal group. However, the TLR4 mRNA after ICH significantly increased starting from 6 hours after ICH (*P* <.05), peaked on 3 days after ICH (*P* < .05), and then decreased on 7 days after ICH, but still maintained at a higher level compared with saline control (*P* < .05). #: compared with saline control, *P* < .05; *: compared with 6 hours after ICH, *P* < .05.

TLR4 mRNA levels were detected by Rt-PCR. The low level of TLR4 mRNA was observed in normal rat brain tissue. In the saline control group, the TLR4 mRNA slightly increased, but was not significantly different from normal group. However, the TLR4 mRNA after ICH significantly increased starting from 6 hours after ICH (*P* < .05), peaked on the 3rd day after ICH, and then decreased on the 7th day after ICH (*P* < .05), but still maintained a higher level compared with saline control (*P* < .05, Figure 1).

### 3.2. Expression of TLR4 Protein Increased in Brain Tissue after ICH

**Figure 2:**
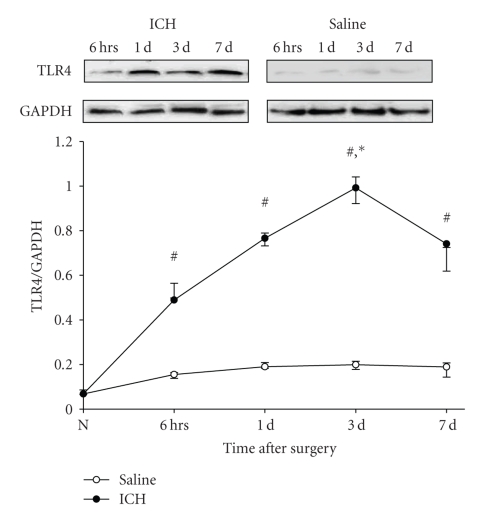
*The temporal changes of TLR4 protein in brain tissue after ICH*. The expression of TLR4 protein was measured in brain tissue by the method of Western Blots using specific antibody for TLR4. The signals were quantified by scanning densitometry and computer-assisted image analysis and expressed as the ratio of TLR4 protein to GAPDH. The results showed that low level of TLR4 protein was detectable in the saline control group. The expression of TLR4 significantly increased starting from 6 hours after ICH (*P* < .05), peaked on 3 days after ICH, and then decreased but still maintained at a high level on 7 days after ICH (*P* < .05). #: compared with saline control, *P* < .05; *: compared with 6 hours after ICH, *P* < .05.

To investigate the increased TLR4 at the protein level, the expression of TLR4 protein was measured in brain tissue by the method of Western Blots using specific antibodies for TLR4. Our results showed that a low level of TLR4 protein was detectable in the saline control group. The expression of TLR4 significantly increased starting from 6 hours after ICH (*P* < .05), peaked on the 3rd day after ICH, and then decreased but still maintained at a high level on the 7th day after ICH (*P* < .05, Figure 2). This observation of TLR4 protein was consistent with the temporal profile of the TLR4 mRNA after ICH.

### 3.3. Distribution of TLR4 in Brain Subjected to ICH

**Figure 3:**
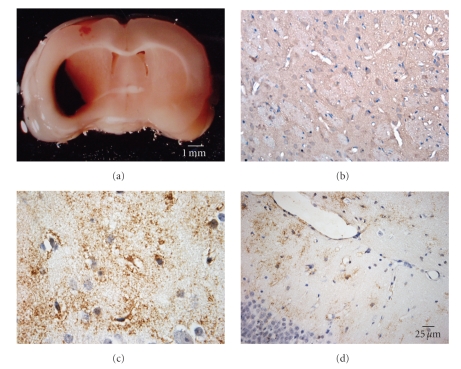
T*he distribution of TLR4 protein in brain tissue after ICH*. Immunohistochemistry staining for TLR4 was performed on brain sections. Figure 3(a) showed the brain section of the model of ICH. Results from the immunohistochemistry staining showed that TLR4 immunoreactivity was not detectable in brain tissue from normal and sham control (Figure 3(b)). However, immunoreactivity for TLR4 was consistently demonstrated in peri-hemorrhage areas, the hippocampus, cortex, thalamic nuclei, and some white matter tracts (Figures 3(c) and 3(d)).

To explore the distribution of TLR4 protein, the TLR4 was detected by immunohistochemistry (IHC). Results showed that TLR4 immunoreactivity was not detectable in brain tissue from normal and sham control. However, immunoreactivity for TLR4 was consistently demonstrated in peri-hemorrhage areas, the hippocampus, cortex, thalamic nuclei, and some white matter tracts (Figure 3).

### 3.4. Activation of TLR4-Mediated NF-Kappa-B Signaling

**Figure 4:**
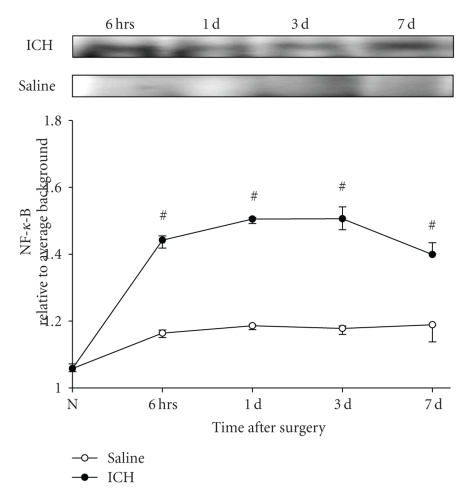
*NF*κ*B activation in the brain in the rats subjected to ICH*. Nuclear proteins were isolated from brain tissues and the NF*κ*B binding activity was examined by the method of EMSA. The signals were quantified by scanning densitometry and computer-assisted image analysis. The results were expressed as the ratio of the integrated density volume (IDV) of NF*κ*B to the average IDV of background. The results showed that the activity of NF*κ*B increased at 6 hours after ICH (*P* < .05), peaked on 3 days after ICH and maintained a high level on 7 days after ICH (*P* < .05). #: compared with saline control, *P* < .05).

**Figure 5:**
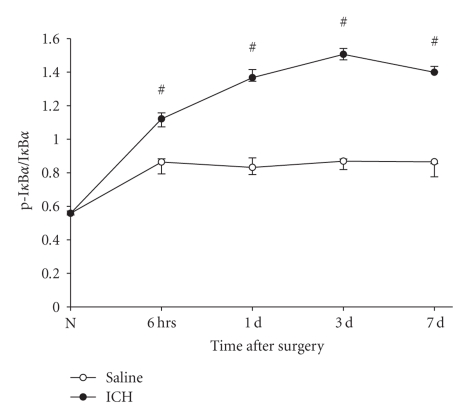
*The increased phosphorylation of inhibitor of* *kappa*-*B* *α* (*p*-*I* *κ* *B* *α*)*in brain after ICH*. The expression of p-I*κ*B in brain tissue was measured by the method of Western Blots using specific antibodies for p-I*κ*B*α* and I*κ*B*α*. The signals were quantified by scanning densitometry and computer-assisted image analysis and expressed as the ratio of p-I*κ*B*α* to I*κ*B*α*. The results showed that the p-I*κ*B increased at 6 hours after ICH (*P* < .05), peaked on 3 days after ICH and maintained a high level on 7 days after ICH (*P* < .05). #: compared with saline control, *P* < .05.

Activation of TLR4 induces the phosphorylation of inhibitor of kappa-B (I*κ*B), which dissociates I*κ*B with NF-kappa-B (NF*κ*B). Then, NF*κ*B translocates into the nucleus and activates and regulates the transcription of genes related to inflammatory responses. To detectd the activation of TLR4-mediated NF*κ*B signaling, the phosphorylation of inhibitor of kappa-B (p-I*κ*B) and activity of NF*κ*B were measured by methods of Western Blots and EMSA. Results showed that the p-I*κ*B increased at 6 hours after ICH (*P* < .05), peaked on 3 days after ICH, and maintained a high level on 7 days after ICH (*P* < .05, Figure 5). As expected, the activity of NF*κ*B was also increased in the same manner as that of the p-I*κ*B (Figure 4).

## 4. Discussion

Intra-cerebral hemorrhage (ICH) represents 10–15% of all strokes, and occurs with increasing frequency as a complication of the thrombolytic treatment of ischemic stroke [1]. The mortality of ICH is higher than that of ischemic stroke. Only 31% are functionally independent at 3 months. Only 38% of the patients survive the 1st year [10]. Unfortunately, the method for treatment of this disease and protection of the brain tissue after ICH are not effective. Mass effect, ischemia, and toxicity of blood components after ICH induce brain tissue damage. In addition, there is increasing evidence that inflammatory processes are involved in cerebrovascular events. After ICH, inflammatory mediators from the blood might enter to the brain and induce an inflammatory reaction and the brain cells itself are also capable of producing many of these agents [11]. The inflammatory responses are responsible for the ICH induced brain injury, which could provide new therapeutic targets for ICH. Various constituents of the inflammatory response, including adhesion molecules, cytokines, leukocytes, immunoglobulins, and complement, may be important in the pathogenesis of ICH. However, how the inflammatory responses is activated and regulated in the brain tissue after ICH is unclear.

 Transcription factor nuclear factor-kappa-B (NF*κ*B) is an important nuclear transcription factor, which initiates transcription of genes associated with immune responses and inflammation [12, 13] and also plays a key role in regulating inflammation in brain pathologies. It has been reported that NF*κ*B increased in the region of perihematoma after ICH [14]. NF*κ*B activation occurs within minutes and persists for at least a week in response to ICH, which is associated with the expression of selected target genes [15]. Activation of NF*κ*B initiates inflammatory responses and contributes to the pathobiology of perilesional cell death after ICH [16]. Inhibition of NF*κ*B activity has been shown to have a therapeutic effect on experimental ICH, such as reduce inflammation, behavioral dysfunction, and neuronal damage produced by ICH [17]. The knowledge about how NF*κ*B is activated in the brain subjected to ICH is critical for seeking a new neuroprotective method for ICH. 

 Toll-like receptors (TLRs) are a family of signal transduction molecules and play a critical role in the induction of innate and adaptive immunity [4]. TLR-mediated signaling pathways mainly stimulate the activation of NF*κ*B [12, 13]. TLR4, the first mammalian TLR recognized, has been reported to be involved in several central nervous (CNS) system diseases, such as inflammatory or autoimmune CNS diseases and cerebral ischemic injury [7, 8]. Although previous reports indicated that the activation of NF*κ*B was attributed to the oxidative stress or glutamate receptor activation induced by red blood cells and/or plasma constituents [17], very few studies have been directed to investigate the role and the relationship between TLR4-mediated NF*κ*B signaling and ICH. 

 In the present study, we established the rat model of experimental ICH and detected the TLR4 mRNA and protein. Our data showed that transcription of TLR4 as well as the expression of TLR4 protein after ICH significantly elevated in brain tissue. These changes started as early as 6 hours after the ICH insult and maintained at a high level for 7 days afterward. The TLR4 protein, detected by immunohistochemistry, was consistently distributed in peri-hemorrhage areas, the hippocampus, cortex, thalamic nuclei, and some white matter tracts. These data indicated that increased expression of TLR4 may be related to the triggering of the inflammatory responses in the brain tissue after ICH.

 Recent studies demonstrated that ICH initiates inflammatory responses, which are associated with secondary growth of hemorrhage and cell death [18]. It is now believed that TLR4-mediated-NF*κ*B pathway activates and regulates immunological and inflammatory responses. To investigate the mechanisms underlying the increased inflammatory responses in the brain after ICH, in the present study, we evaluated the activation of TLR4-mediated NF*κ*B signaling by detecting the p-I*κ*B and the activity of NF*κ*B in hemorrhagic brains. NF*κ*B is an important transcription factor downstream in the TLR4-mediated signaling pathway. Activation of TLR4 stimulates I*κ*B*α* phosphorylation and degradation, resulting in nuclear translocation of NF*κ*B, which initiates transcription of genes associated with innate immune responses and inflammation [5, 6]. We observed that the levels of phosphorylation of I*κ*B and the activity of NF*κ*B were significantly increased in brain tissue after ICH. The temporal profile of the expression of p-I*κ*B and the activation of NF*κ*B was similar to that of the expression of TLR4. Our data suggest that ICH stimulates activation of the TLR4/NF*κ*B signaling pathway, which is consistent with previous reports that activation of NF*κ*B is regulated through the TLR4-mediated signaling pathway. TLR4 plays an important role in the recognition of microbial components [19, 20] and can also recognize endogenous molecules such as the degradation products of macromolecules, products of proteolytic cascades, intracellular components of ruptured cells, and products of genes that are activated by inflammation. For instance, HSP70, HSP32, HSP27, and enzymes involved in oxidative stress are found to be upregulated in the brain after ICH, which may be produced by injured neuronal cells or blood cells [21]. It may be speculated that these molecules produced in damaged brain or came from blood cells and constituents of plasma server as endogenous TLR4 ligands, activate TLR4-mediated NF*κ*B signaling, and initiate the inflammatory responses in IHC brain. However, the specific endogenous ligands for TLR4 in hemorrhagic brain have not been determined and need to be identified through further research.

 In conclusion, the present study demonstrated the temporal profile of the activation of TLR4-mediated NF*κ*B signaling in brain tissue in a rat model of experimental ICH, which suggests that TLR4-mediated NF*κ*B signaling participates in the pathogenesis of ICH, which may be a therapeutic target for the prevention of ICH-induced brain damage.
